# Overexpression of the primary sigma factor gene *sigA* improved carotenoid production by *Corynebacterium glutamicum*: Application to production of β-carotene and the non-native linear C50 carotenoid bisanhydrobacterioruberin

**DOI:** 10.1016/j.meteno.2017.01.001

**Published:** 2017-01-13

**Authors:** Hironori Taniguchi, Nadja A. Henke, Sabine A.E. Heider, Volker F. Wendisch

**Affiliations:** a Genetics of Prokaryotes, Faculty of Biology & CeBiTec, Bielefeld University, P.O. Box 100131, 33501 Bielefeld, Germany; b Synthetic bioengineering, Department of Biotechnology, Osaka University, Yamadaoka 2-1, Suita, 565-0871 Osaka, Japan; c GSK Vaccines S.r.I., Siena 53100, Italy

**Keywords:** WT, wild type, CDW, cell dry weight, MEP, methylerythritol 4-phosphate, PCA, protocatechuic acid, OD, optical density, HPLC, high performance liquid chromatography, BHT, Butylhydroxytoluol, BABR, bisanhydrobacterioruberin, Decaprenoxanthin (PubChem CID: 6443309), Lycopene (PubChem CID: 446925), β-carotene (PubChem CID: 5280489), Bisanhydrobacterioruberin (PubChem CID: 10930540), Thiamine (PubChem CID: 1130), Protocatecuic acid (PubChem CID: 72)

## Abstract

*Corynebacterium glutamicum* shows yellow pigmentation due to biosynthesis of the C50 carotenoid decaprenoxanthin and its glycosides. This bacterium has been engineered for production of various non-native cyclic C40 and C50 carotenoids such as β-carotene, astaxanthin or sarcinaxanthin. In this study, the effect of modulating gene expression more broadly by overexpression of sigma factor genes on carotenoid production by *C. glutamicum* was characterized. Overexpression of the primary sigma factor gene *sigA* improved lycopene production by recombinant *C. glutamicum* up to 8-fold. In *C. glutamicum* wild type, overexpression of *sigA* led to 2-fold increased accumulation of the native carotenoid decaprenoxanthin in the stationary growth phase. Under these conditions, genes related to thiamine synthesis and aromatic compound degradation showed increased RNA levels and addition of thiamine and the aromatic iron chelator protocatechuic acid to the culture medium enhanced carotenoid production when *sigA* was overexpressed. Deletion of the gene for the alternative sigma factor SigB, which is expected to replace SigA in RNA polymerase holoenzymes during transition to the stationary growth phase, also increased carotenoid production. The strategy of *sigA* overexpression could be successfully transferred to production of the non-native carotenoids β-carotene and bisanhydrobacterioruberin (BABR). Production of the latter is the first demonstration that *C. glutamicum* may accumulate a non-native linear C50 carotenoid instead of the native cyclic C50 carotenoid decaprenoxanthin.

## Highlights

• Overexpression of the primary sigma factor gene *sigA* enhanced carotenoid production.
• Enhanced production of carotenoids in the absence of alternative sigma factor SigB.
• Production of the linear C50 carotenoid bisanhydrobacterioruberin established.

## Graphical abstract

**Figure:**
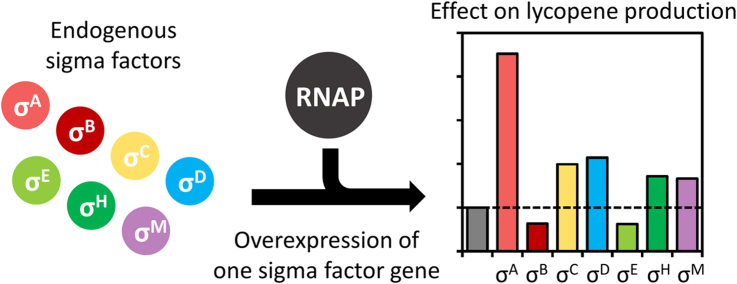

## 1 Introduction

Carotenoids are natural pigments which show various colors from yellow to red depending on their chemical structures (Britton et al., 2004). Because of versatile applications especially in food and feed industries, the demand for bio-based carotenoid production is increasing (Breithaupt, 2007, Scotter, 2011). In order to increase the production and efficiency, metabolic engineering has been applied in the natural carotenoid producers as well as in non-carotenogenic organisms (Ausich, 2009, Ye and Bhatia, 2012). The pathways and related enzymes for carotenogenesis are well understood. First, IPP (Isopentenyl pyrophosphate) and DMAPP (Dimethylallyl pyrophosphate) are synthesized either by the mevalonate (MEV) pathway or the methylerythritol phosphate (MEP) pathway (Chang et al., 2013). IPP and its isomer DMAPP are condensed to geranylgeranyl pyrophosphate (GGPP), of which lycopene is synthesized (Moise et al., 2014). Lycopene is a red C_40_ carotenoid that serves as precursor for the synthesis of other C40 and C50 carotenoids such as β-carotene, lutein or astaxanthin (Heider et al., 2014a, Misawa and Shimada, 1997).

*Corynebacterium glutamicum* is a non-pathogenic bacterium that is used for several decades for the million-ton-scale amino acid production, especially for L-glutamate and L-lysine (Eggeling and Bott, 2005). Furthermore, this bacterium has been engineered for production of various industrially relevant compounds (Wendisch, 2014). *C. glutamicum* naturally produces the C50 carotenoid decaprenoxanthin (Krubasik et al., 2001). Recently, *C. glutamicum* strains overproducing lycopene and other carotenoids including β-carotene and astaxanthin have been developed (Heider et al., 2014b, Heider et al., 2014c, Heider et al., 2012, Henke et al., 2016). *C. glutamicum* is a suitable production host for carotenoids; the volumetric productivity for astaxanthin of up to 0.4 mg L^−1^ h^−1^ is comparable to current algae-based production (Henke et al., 2016) and high cell density fermentation with up to 100 g cell dry weight (CDW) L^−1^ is established (Knoll et al., 2007).

Production strain development may involve regulatory engineering, e.g. of sigma factor genes (Tripathi et al., 2014). Sigma factors are important for promoter recognition and transcription initiation as one of the subunits of RNA polymerase holoenzyme (Gruber and Gross, 2003). Bacteria often possess multiple sigma factor genes which encode primary and alternative sigma factors (Feklístov et al., 2014). Depending on the sigma factor targeted, sigma factor engineering may alter the transcription profile globally or may affect a subset of genes that share similar functions. For example, deletion of the general stress sigma factor gene *rpoS* improved putrescine production by recombinant *E. coli* (Qian et al., 2009). Overexpression of the nitrogen starvation sigma factor gene *rpoN* in *E. coli* improved heterologous expression of polyketide and non-ribosomal peptide biosynthetis gene clusters of *Streptomyces rimosus*, which may be due to the presence of RpoN promoter sequences in the majority of them (Stevens et al., 2013). *E. coli* strains with improved production characteristics such as ethanol tolerance could be selected from a population overexpressing randomly mutated RpoD (Alper and Stephanopoulos, 2007). In *Synechocystis* sp. PCC 6803, overexpression of alternative sigma factor gene *sigE* improved production of polyhydroxybutyrate (Osanai et al., 2013). In *C. glutamicum*, which possesses seven sigma factors SigA, SigB, SigC, SigD, SigE, SigH and SigM (Pátek and Nešvera, 2011, Toyoda and Inui, 2016), overexpression of *sigH* resulted in overproduction of riboflavin and of flavin mononucleotide (FMN) when combined with overexpression of the endogenous gene encoding bifunctional riboflavinkinase/FMN adenyltransferase (Taniguchi and Wendisch, 2015). In this study, the effect of overexpression of sigma factor genes on carotenoid production was elucidated and overexpression of the general sigma factor gene *sigA* was shown to increase overproduction of the C40 carotenoids lycopene and β-carotene as well as the C50 carotenoids decaprenoxanthin and bisanhydrobacterioruberin.

## 2 Material and methods

### 2.1 Bacterial strains, plasmids and oligonucleotides

The strains, plasmids and oligonucleotides (Metabion, Martinsried, Germany) used in this work are listed in Table 1. Plasmids were constructed based on pVWEx1 or pEKEx3, which are both IPTG inducible expression vectors for *E. coli* and *C. glutamicum* (Peters-Wendisch et al., 2001, Stansen et al., 2005). Plasmid and strain construction was performed as described previously (Taniguchi and Wendisch, 2015). Briefly, the DNA fragment of the target gene was amplified with the respective oligonucleotide pairs in Table 1, and inserted into pVWEx1, alternatively pEKEx3, by Gibson assembly (Gibson et al., 2009). *E. coli* DH5α was used for cloning. The sequence of inserted DNA fragments was confirmed by sequencing (CeBiTec Sequencing Core Facility, Bielefeld, Germany). *C. glutamicum* competent cells were transformed by electroporation at 2.5 kV, 200 Ω, and 25 μF (Eggeling and Bott, 2005, van der Rest et al., 1999).

**Table 1:** Bacterial strains, plasmids and oligonucleotides used in this study.

**Bacterial strain**	**Relevant characteristic**	**Reference or source**
*C. glutamicum*		
WT	Wild-type, ATCC 13032	ATCC
WTΔ*sigB*	*sigB* deletion mutant of WT ATCC 13032	this study
LYC5	LYC3-P_*tuf*_-*dxs* derivatives with insertion of *crtEBI* operon under the control of P_*tuf*_ promoter integrated into the *cgp*2 cured region between cg1745 and cg1753	(Henke et al., 2016)
LYC5Δ*sigB*	*sigB* deletion mutant of LYC5	this study
BETA3	LYC5 derivatives with insertion of *crtY* from *P. ananatis* under the control of P_*tuf*_ promoter integrated into the *cgp*2 cured region between cg1745 and cg1753	(Henke et al., 2016)
BETA3Δ*sigB*	*sigB* deletion mutant of BETA3	this study
*E. coli*		
*E. coli* DH5α	F−*thi*−1 *endA*1 *hsdR*17(r−, m−) *supE*44 Δ*lacU*169 (Φ80*lacZ*ΔM15) *recA*1 *gyrA*96 *relA*1	Bethesda Research Laboratories
		
**Plasmid**	**Relevant characteristic**	**References**
pVWEx1	KanR; *E. coli*-*C. glutamicum* shuttle vector for regulated gene expression (P_*tac*_, *lacI*^q^, pCG1 oriVCg)	(Peters-Wendisch et al., 2001)
pVWEx1-*sigA*	KanR, pVWEx1 with *sigA* from *C. glutamicum* WT	this study
pVWEx1-*sigB*	KanR, pVWEx1 with *sigB* from *C. glutamicum* WT	this study
pVWEx1-*sigC*	KanR, pVWEx1 with *sigC* from *C. glutamicum* WT	this study
pVWEx1-*sigD*	KanR, pVWEx1 with *sigD* from *C. glutamicum* WT	this study
pVWEx1-*sigE*	KanR, pVWEx1 with *sigE* from *C. glutamicum* WT	this study
pVWEx1-*sigH*	KanR, pVWEx1 with *sigH* from *C. glutamicum* WT	(Taniguchi and Wendisch, 2015)
pVWEx1-*sigM*	KanR, pVWEx1 with *sigM* from *C. glutamicum* WT	this study
pVWEx1-*lbtBC*	KanR, pVWEx1 with *lbtBC* from *Dietzia* sp. CQ4	this study
pEKEx3	SpecR; *E. coli*-*C. glutamicum* shuttle vector for regulated gene expression (P_*tac*_, *lacI*^q^, pCG1 oriVCg)	(Stansen et al. 2005)
pEKEx3-*sigA*	SpecR, pEKEx3-*sigA* from *C. glutamicum* WT	(Taniguchi and Wendisch, 2015)
pK19mobsacB	KmR; *E.* coli-C*. glutamicum* shuttle vector for construction of insertion and deletion mutants in *C. glutamicum* (pK18 *oriVEc sacB lacZα*)	(Schäfer et al., 1994)
pK19mobsacB-Δ*sigB*	KmR; *E.* coli-C*. glutamicum* shuttle vector for construction of deletion mutant Δ*sigB* in *C. glutamicum*	this study
		
**Oligonucleotide**	**Sequence (5′-3′)**	**References**
*sigA*-fwd	GCCTGCAGGTCGACTCTAGAG***GAAAGGAGG***CCCTTCAG**ATG**GTAGAAAACAACGTAGCAAAAAAGACGGTCG	(Taniguchi and Wendisch, 2015)
*sigA*-rev	CGGTACCCGGGGATCTTAGTCCAGGTAGTCGCGAAGGACCTG	(Taniguchi and Wendisch, 2015)
*sigB*-fwd	GCCTGCAGGTCGACTCTAGAG***GAAAGGAGG***CCCTTCAG**ATG**ACAGCACCGTCCACGCAG	(Taniguchi and Wendisch, 2015)
*sigB*-rev	CGGTACCCGGGGATCTTACTGGGCGTACTCACGAAGACGTG	(Taniguchi and Wendisch, 2015)
*sigC*-fwd	GCCTGCAGGTCGACTCTAGAG***GAAAGGAGG***CCCTTCAG**GTG**AAGTCAAAAGAGCGTAACGACGC	(Taniguchi and Wendisch, 2015)
*sigC*-rev	CGGTACCCGGGGATCCTAACCTTGGGCGGATTTGCCATCTTCG	(Taniguchi and Wendisch, 2015)
*sigD*-fwd	GCCTGCAGGTCGACTCTAGAG***GAAAGGAGG***CCCTTCAG**TTG**GCTGATACTGAGCGCGAGCTC	(Taniguchi and Wendisch, 2015)
*sigD*-rev	CGGTACCCGGGGATCTTACTTGTTCTCCTGCTGCTCAAGTGTGCTTC	(Taniguchi and Wendisch, 2015)
*sigE*-fwd	GCCTGCAGGTCGACTCTAGAG***GAAAGGAGG***CCCTTCAG**ATG**ACTTATATGAAAAAGAAGTCCCGAGATGACGCAC	(Taniguchi and Wendisch, 2015)
*sigE*-rev	CGGTACCCGGGGATCTTAGTGGGTTGGAACCAACAAAGAAACTTCCTCG	(Taniguchi and Wendisch, 2015)
*sigH*-fwd	GCCTGCAGGTCGACTCTAGAG***GAAAGGAGG***CCCTTCAG**ATG**GCTGAAAACCGAACCGGCAC	(Taniguchi and Wendisch, 2015)
*sigH*-rev	CGGTACCCGGGGATCTTATGCCTCCGAATTTTTCTTCATGTCGGGATG	(Taniguchi and Wendisch, 2015)
*sigM*-fwd	GCCTGCAGGTCGACTCTAGAG***GAAAGGAGG***CCCTTCAG**ATG**ACAGTACTGCCTAAAAACCATGACCTAAGC	(Taniguchi and Wendisch, 2015)
*sigM*-rev	CGGTACCCGGGGATCTCAGTTGCTTTCGCACTGTATGGAGCC	(Taniguchi and Wendisch, 2015)
*lbtBC-fwd*	CATGCCTGCAGGTCGACTCTAGAG***GAAAGGAGG***CCCTTCAG**ATG**ACCTCCCTGTACACCAC	this study
*lbtBC-rev*	ATTCGAGCTCGGTACCCGGGGATCTTAGGACCACACCAGCACGGA	this study
*sigB-A*	TGCCTGCAGGTCGACTCTAGAGATTGCTGAGCTGCGCATCT	this study
*sigB-B*	GGGTAGGTGATTTGAATTTGTCGTGGACGGTGCTGTCAT	this study
*sigB-C*	ACAAATTCAAATCACCTACCCGAGCGCGCATCACGTCTT	this study
*sigB-D*	ATTCGAGCTCGGTACCCGGGGATCTCCAAACTCAATTTATGCCGCT	this study
*sigB-E*	ATTGTTGGAGCCATCGAT	this study
*sigB-F*	ACTGCTCAAGGCGTTCT	this study
*sigB seq1*	AGATTGCACAAGGTTTAC	this study
*sigB seq2*	AGAAAACTTCCCCGTATC	this study

Underlined sequences represent the overlap region with a vector plasmid; sequences in bold italic represent a ribosome binding site; sequences in bold represents the translational start codon.

### 2.2 Chromosomal deletion of *sigB* in *C. glutamicum*

For targeted deletion of *sigB*, the suicide vector pK19*mobsacB* was used (Schäfer et al., 1994). Genomic regions flanking *cg2102* were amplified from the genomic DNA of *C. glutamicum* WT ATCC 13032 using oligonucleotide pairs *sigB*-A/B and *sigB*-C/D (Table 1), respectively. Afterwards, the purified PCR products were linked and cloned into *Bam*HI digested pK19*mobsacB* via Gibson Assembly (Gibson et al., 2009). The resulting deletion vector pK19*mobsacB*-Δ*sigB* (Table 1) was confirmed via sequencing. Introduction of pK19*mobsacB*-Δ*sigB* into *C. glutamicum* was carried out via trans conjugation with *E. coli* S17-1 (Schäfer et al., 1994). Deletion of *sigB* via two-step homologous recombination as well as the selection for the first and second recombination events were carried out as described previously (Eggeling and Bott, 2005). Successful deletion of *sigB* was verified by PCR analysis of the constructed mutant using oligonucleotide pair *sigB-*E/F (Table 1) and sequencing of the PCR product.

### 2.3 Medium, growth condition and growth rate comparison

As far as not mentioned specifically, *C. glutamicum* was precultured in LB medium (Sambrook, 2001) with 56 mM of glucose overnight, washed once with CGXII medium (Eggeling and Bott, 2005) without carbon source and inoculated in CGXII with 222 mM of glucose at initial optical density (OD) (λ=600 nm) of 1. The OD was measured with UV-1202 spectrophotometer (Shimadzu, Duisburg, Germany) with suitable dilutions. When appropriate, 25 μg/mL of kanamycin and IPTG were added as indicated in the text. Growth experiment with Biolector^®^ cultivation system (m2pLabs, Baesweiler, Germany) was performed in 1 mL of CGXII with 222 mM of glucose using FlowerPlate^®^ (m2pLabs, Baesweiler, Germany) at 30 °C, 1100 rpm. Growth experiment with flask was performed in 50 mL of CGXII 222 mM of glucose using 500 mL of baffled flask at 30 °C, 120 rpm. For growth rate calculation, cell growth was monitored online every 10 min for 48 h with Biolector^®^. Maximum growth rate μ (h^−1^) was calculated from 20 measuring points of arbitrary unit of the backscattering light (620 nm). Plate image was scanned with Perfection V750-M Pro scanner (Epson, Ludwigshafen am Rhein, Germany). Consumption of glucose was tested with Diabur 5000 glucose test stripes (Roche Diagnostics, Mannheim, Germany) with a detection limit of 0.005% glucose.

### 2.4 Carotenoid extraction and quantification

For quantification of lycopene, decaprenoxanthin and β-carotene, 200–1000 μL of the cell culture was centrifuged and washed once with 500 μL of CGXII without carbon source. The cell pellet was frozen and stored at −20 °C until further use. The cell pellet was resuspended in 400–800 μL of acetone and carotenoids were extracted for 60 min at 50 °C and 1400 rpm. The supernatant was separated by centrifugation and absorption spectrum from 400 nm to 550 nm was measured by UV-1800 spectrophotometer (Shimadzu, Duisburg, Germany). The spectrum was normalized by the absorbance at wavelength of 550 nm and the maximum absorbance in the spectrum was used for quantification of each carotenoid (474 nm for lycopene, 440 nm for decaprenoxanthin, 454 nm for β-carotene). The quantification of bisanhydrobacterioruberin (BABR) was performed by high performance liquid chromatography (HPLC) with the Agilent 1200 series system (Agilent Technologies Sales & Services GmbH & Co. KG, Waldbronn). For HPLC analysis, pigments were extracted with 800 μL methanol: acetone (7:3) containing 0.05% BHT (Butylhydroxytoluol) at 60 °C for 15 min with careful vortexing every 5 min. The supernatant was separated by centrifugation and used for analysis. 50 μL of the sample was separated with a column system consisting of a precolumn (LiChrospher 100 RP18 EC-5, 40×4 mm, CS-Chromatographie, Langerwehe, Germany) and a main column (LiChrospher 100 RP18 EC-5, 125×4 mm, CS-Chromatographie) with methanol (A) and methanol/water (9:1) (B) as mobile phase. The following gradient was used at a flow rate of 1.5 mL/min; 0 min B: 0%, 10 min B: 100%, 32.5 min B: 100%. The UV/visible (Vis) spectrum was recorded with a diode array detector (DAD) and the amount of carotenoids was quantitated by the integration of the extracted wavelength chromatogram at 471 nm and by the analysis of the appropriate UV/Vis profiles. For quantification of lycopene and β-carotene, pure standards (Sigma-Aldrich, Germany) were used. Due to the lack of commercially available standards for decaprenoxanthin and BABR, the concentrations of these are given as β-carotene equivalents using standardization with pure β-carotene. The amount of each carotenoid production was normalized against cell weight/optical density.

### 2.5 Transcriptome analysis of the *sigA* overexpressing strain using DNA microarray

*C. glutamicum* strains WT(pVWEx1) and WT(pVWEx1-*sigA*) were cultured in LB medium with 56 mM of glucose and 25 μg/mL of kanamycin and inoculated into CGXII medium with 222 mM of glucose and 25 μg/mL of kanamycin for adaptation to glucose as carbon source. Cells were cultured overnight and inoculated into 50 mL of CGXII medium with 222 mM of glucose, 50 μM of IPTG and 25 μg/mL of kanamycin at the initial OD of 1. Cells were harvested 8 h after inoculation in the early exponential growth phase (OD between 6 and 8) and after 24 h in the stationary phase. RNA isolation was performed as described previously (Wendisch, 2003). The purified RNA was analyzed by spectrophotometer (NanoDrop) for quantity and gel electrophoresis for quality. The RNA sample was stored at −80 °C until further use. cDNA synthesis as well as DNA microarray hybridization were performed as described previously (Netzer et al., 2004, Polen et al., 2007). Normalization and evaluation of the data was done with the software package EMMA 2 (Dondrup et al., 2009). Genes which expression was upregulated or downregulated in WT(pVWEx1-*sigA*) were taken into account for further analysis (FDR<0.05, M-value>1 for upregulation, M-value<−1 for downregulation).

## 3 Results

### 3.1 Overexpression of *sigA* improved production of lycopene by *C. glutamicum*

**Fig. 1:**
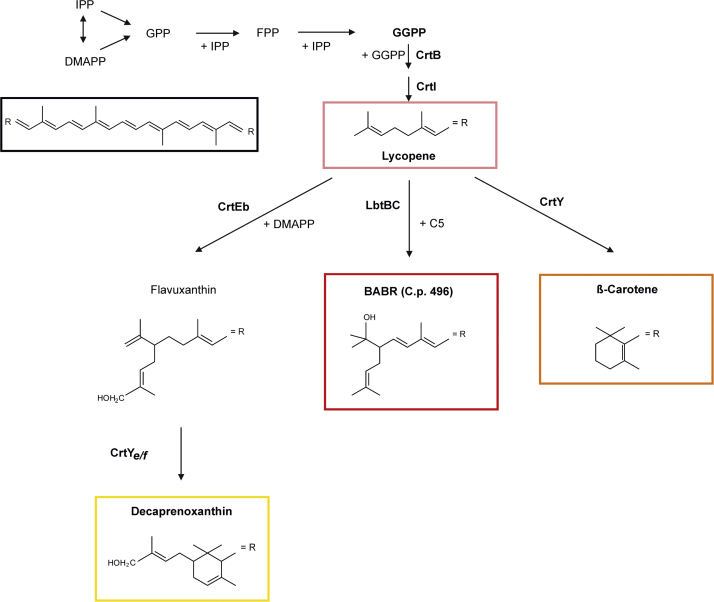
**Scheme of carotenogenesis in*****C. glutamicum*****and engineered pathway leading to non-native carotenoids.** The native pathway of decaprenoxanthin biosynthesis initiating with isopentenyl pyrophosphate via lycopene is depicted next to the introduced pathways leading to β-carotene and bisanhydrobacterioruberin (BABR), respectively. CrtEb, endogenous lycopene elongase; CrtYe/f, endogenous carotenoid C45/C50 ɛ-cyclase; LbtBC, lycopene elongase from *Dietzia* sp. CQ4; CrtY, lycopene cyclase from *Pantoea ananatis*.

**Fig. 2:**
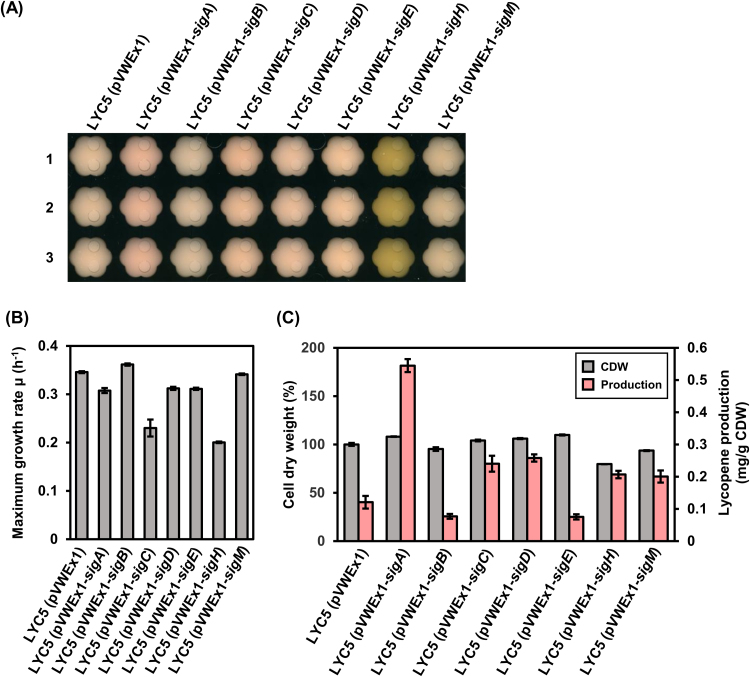
**Overexpression of sigma factor genes in the lycopene producing strain LYC5.** FlowerPlate image of cultures (A) of biological triplicates after 48 h of cultivation are shown in rows 1–3. Maximum growth rates (B), cell weights and lycopene production (C) are given as means of biological triplicates with standard deviations. Lycopene production was calculated based on the absorbance of carotenoid extract at wavelength of 474 nm and normalized by cell weight used for extraction. Cell dry weight is shown as percentages of the control strain LYC5(pVWEx1).

Lycopene is a central intermediate of carotenogenesis in *C. glutamicum* (Fig. 1). In the lycopene producing strain LYC5 sigma factor genes were overexpressed and their influence on lycopene production was evaluated in Biolector microscale cultivations. The transformants were grown in minimal medium with glucose as carbon source and sigma factor gene expression from pVWEx1 was induced with 50 µM IPTG. Overexpression of sigma factor genes influenced the maximum growth rate differently (Fig. 2). Overexpression of *sigA*, *sigD* and *sigE* led to a slight decrease of the growth rate, and overexpression of *sigB* and *sigM* did not influence the growth rate. On the other hand, overexpression of *sigC* and *sigH* slowed cell growth considerably. Overexpression of sigma factor genes influenced the final biomass only to a small extent (<±10%) except for *sigH* (−20%). Overexpression of *sigH* in LYC5 led to accumulation of riboflavin in the supernatant as reported previously for overexpression of *sigH* in *C. glutamicum* wild type (Taniguchi and Wendisch, 2015). Overexpression of *sigA* led to more reddish colored cells compared to the control strain, LYC5(pVWEx1) (Fig. 2). Quantification of lycopene showed that overexpression of *sigC*, *sigD*, *sigH* and *sigM* led to moderately increased lycopene production, while overexpression of *sigB* and *sigE* decreased production (Fig. 2). Furthermore, *sigA* overexpressing cells accumulated about 3.5-fold more lycopene than the control strain (Fig. 2).

**Fig. 3:**
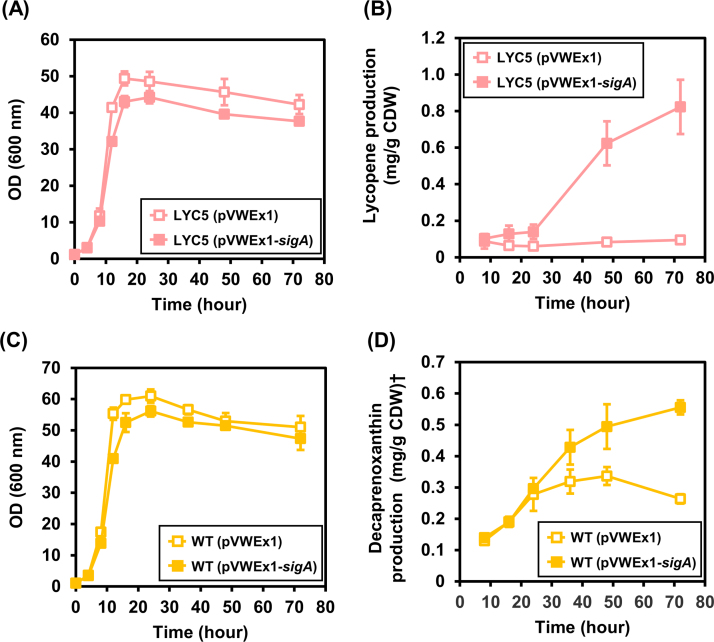
**Effect of*****sigA*****overexpression on accumulation of lycopene (A) and decaprenoxanthin (B) during growth.** Growth (A) and lycopene production (B) of strains LYC5(pVWEx1) and LYC5(pVWEx1-*sigA*) are shown. Growth (C) and decaprenoxanthin production (D) of strains WT(pVWEx1) and WT(pVWEx1-*sigA*) are shown. Lycopene and decaprenoxanthin production were calculated based on the absorbance of carotenoid extracts at 474 nm for lycopene and 440 nm for decaprenoxanthin and were normalized by cell weight used for extraction. Cultivations were performed in baffled flasks. OD and carotenoid production are given as means of biological triplicates with standard deviations. †Decaprenoxanthin production was calculated as β-carotene equivalent.

To verify the effect of *sigA* overexpression on lycopene production, growth and lycopene accumulation was followed for 72 h during different growth phases using shake flasks. Cells reached the stationary growth phase (Fig. 3A) and glucose was consumed completely after 24 h. For the first 24 h of cultivation, lycopene accumulation was significantly but only slightly higher (about 1.5-fold) in the *sigA* overexpressing strain than in the control strain (Fig. 3B). However, the *sigA* overexpressing cells continued to produce lycopene in the stationary growth phase from 24 h to 72 h (Fig. 3B). After 72 h, the *sigA* overexpressing strain produced 0.82±0.15 mg/g CDW lycopene, which was about 8-fold more than the control strain. Thus, overexpression of *sigA* encoding the primary sigma factor of *C. glutamicum* improved lycopene production particularly in the stationary growth phase.

### 3.2 *C. glutamicum* wild type overproduced decaprenoxanthin in the stationary growth phase as consequence of *sigA* overexpression

Overexpression of *sigA* affected lycopene accumulation most in the stationary growth phase (Fig. 3B). To determine if *sigA* overexpression affects biosynthesis of the native carotenoid decaprenoxanthin, *C. glutamicum* WT(pVWEx1-*sigA*) and WT(pVWEx1) were cultivated in glucose minimal medium in shake flasks for 72 h and decaprenoxanthin accumulation during the exponential and stationary growth phases were monitored. During exponential growth and until glucose was exhausted at about 24 h, decaprenoxanthin accumulation hardly differed between both strains (Fig. 3C). However, *C. glutamicum* WT(pVWEx1-*sigA*) continued to produce decaprenoxanthin in the stationary growth phase while WT(pVWEx1) did not (Fig. 3D). After 72 h, decaprenoxanthin production by WT(pVWEx1-*sigA*) was about twice as high as in the control strain (Fig. 3D). Thus, *sigA* overexpression increased decaprenoxanthin biosynthesis by wild type in the stationary growth phase.

### 3.3 Application of *sigA* overexpression to production of the non-native cyclic C40 carotenoid β-carotene

**Fig. 4:**
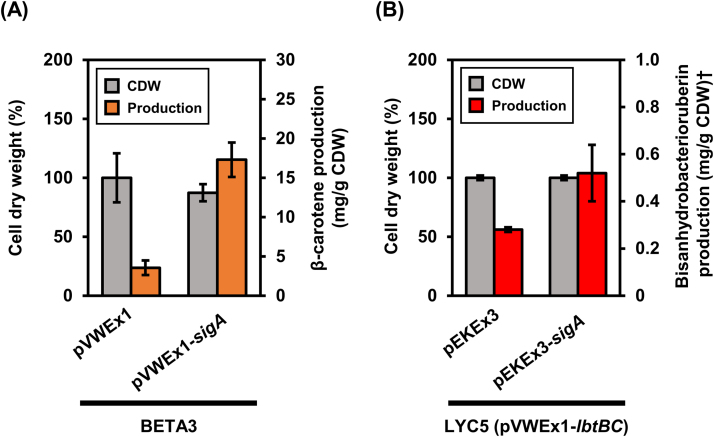
Effect of *sigA* overexpression on production of (A) β-carotene and (B) bisanhydrobacterioruberin (BABR) by recombinant C. glutamicum strains. β-carotene production was calculated based on absorbance of carotenoid extracts at wavelength of 454 nm and normalized by cell weight used for extraction. Bisanhydrobacterioruberin production was calculated based on the HPLC analysis of the dominant peak at 471 nm for carotenoid extracts and normalized by cell weight. Cultivations were performed in baffled flasks. Carotenoid production is given as means of biological triplicates with standard deviations. †BABR production was calculated as β-carotene equivalent.

Lycopene is a precursor for several carotenoids and *sigA* overexpression improved its production. However, as lycopene production is not a reliable indicator of MEP pathway flux at least in *E. coli* (Bongers et al., 2015), we tested if *sigA* overexpression is beneficial for production of a lycopene-derived non-native carotenoid such as β-carotene. *C. glutamicum* WT does not synthesize β-carotene, but the recombinant strain *C. glutamicum* BETA3 produces β-carotene without accumulation of the precursor lycopene (Henke et al., 2016). This strain has been constructed based on *C. glutamicum* LYC5 by genomic integration of *crtY* from *P. ananatis* encoding lycopene cyclase under the control of a strong constitutive promoter (Fig. 1). A comparative growth experiment of *C. glutamicum* BETA3(pVWEx1) and BETA3(pVWEx1-*sigA*) revealed that both strains grew to similar biomass concentrations after glucose was completely consumed (Fig. 4A). In the stationary growth phase, BETA3(pVWEx1-*sigA*) produced 11.9±1.5 mg/g CDW, which is about 3-fold more β-carotene than accumulated by the control strain BETA3(pVWEx1) (Fig. 4A).

### 3.4 Engineering of *C. glutamicum* for overproduction of a non-native linear C50 carotenoid

Carotenoids are believed to span the cytoplasmic membrane and the prevalence of C40 carotenoids may reflect the typical cytoplasmic membrane width (Abbes et al., 2013, Lazrak et al., 1987). Besides the native decaprenoxanthin (Heider et al., 2014c, Krubasik et al., 2001), we showed that recombinant *C. glutamicum* strains overproduced carotenoids of different lengths: linear and cyclic C40 carotenoids such as lycopene and β-carotene (Heider et al., 2014a, Henke et al., 2016) as well as cyclic C50 carotenoids such as sarcinaxanthin (Netzer et al., 2010). Here, we attempted to overproduce a non-native linear C50 carotenoid, namely bisanhydrobacterioruberin (BABR) (Norgård et al., 1970) that differs from the cyclic decaprenoxanthin in length, which may affect membrane integration. To this end, *lbtBC* encoding the lycopene elongase from *Dietzia* sp. CQ4 (Tao et al., 2007) was expressed from the IPTG inducible plasmid pVWEx1 in the lycopene overproducing strain *C. glutamicum* LYC5 (Fig. 1). To apply our finding that *sigA* overexpression improved carotenoid production, the resulting strain LYC5(pVWEx1-*lbtBC*) was transformed with pEKEx3-*sigA*. After cultivation of this strain and an empty vector carrying control strain for 48 h in glucose minimal medium, carotenoids were extracted and quantified. Both *C. glutamicum* LYC5(pVWEx1-*lbtBC*)(pEKEx3) and LYC5(pVWEx1-*lbtBC*)(pEKEx3-*sigA*) accumulated the linear C50 carotenoid bisanhydrobacterioruberin to considerable concentrations. When BABR production was calculated based on a β-carotene standard due to the lack of a standard, *C. glutamicum* LYC5(pVWEx1-*lbtBC*)(pEKEx3) produced 0.28±0.01 mg/g CDW, on the other hand, *C. glutamicum* LYC5(pVWEx1-*lbtBC*)(pEKEx3-*sigA*) produced 0.52±0.12 mg/g CDW bisanhydrobacterioruberin. Thus, plasmid-driven *sigA* overexpression led to 1.8-fold higher BABR production (Fig. 4B).

### 3.5 Global gene expression changes due to *sigA* overexpression

Overexpression of the primary sigma factor gene *sigA* is expected to affect expression of many genes and this effect may differ in the exponential and stationary growth phases. In order to determine which genes are differentially expressed in the exponential and stationary growth phases due to *sigA* overexpression, *C. glutamicum* WT(pVWEx1) and WT(pVWEx1-*sigA*) were cultivated in glucose minimal medium and RNA was extracted in the mid-exponential growth phase (at 8 h) and at the beginning of the stationary growth phase when glucose was exhausted (at 24 h). Global gene expression analysis was determined by DNA microarray analysis (Table 2). The data were deposited at GEO (GSE86866). The analysis revealed significantly decreased RNA levels of 18 genes at 8 h and 32 genes at 24 h (false discovery rate <0.05, M-value<−1) (Table S1, Table S2). Only one gene (cg0612), which is annotated as putative aldo/keto reductase, showed decreased RNA levels both at 8 and 24 h (Table 2). In addition to the overexpressed *sigA* itself, RNA levels for 49 and 64 genes increased significantly at 8 h and 24 h, respectively (false discovery rate<0.05, M-value>1) (Table S1, Table S2). Notably, genes involved in carotenogenesis did not show increased RNA levels upon *sigA* overexpression. At both 8 h and 24 h, RNA levels of nine genes were increased (Table 2): seven genes encoding uncharacterized proteins, the cardiolipin synthase gene *cls* (Nampoothiri et al., 2002) and the catechol 1,2-dioxygenase gene *catA* (Shen et al., 2004). Although catechol 1,2-dioxygenase is not involved in degradation of protocatechuic acid (PCA) (Shen et al., 2004), a medium component added as iron chelator, *catA* is among the genes induced during fast growth with glucose/PCA mixtures (Unthan et al., 2014). Thiamine is not added as medium component, however, thiamine biosynthesis genes were upregulated at both time points: *thiOSG* at 8 h and *thiC* and *thiD1* at 24 h (Table S1, Table S2). Thiamine is necessary as a cofactor for 1-deoxy-D-xylulose-5-phosphate (DXP) synthase (Dxs), which catalyzes the first step of isopentenyl diphosphate (IPP) synthesis in the MEP pathway (Vranová et al., 2013) and in the thiamine biosynthesis pathway (Begley et al., 1999).

**Table 2:** DNA microarray analysis of genes differentially expressed upon *sigA* overexpression after 8 h and after 24 h of cultivation.

Gene ID^a^	Name^a^	Function of protein^a^	At 8 h		At 24 h	
			M-value^b^	FDR^c^	M-value^b^	FDR^c^
cg0612	*dkg*	Putative aldo/keto reductase	−1.2	5.6E−06	−1.2	6.2E−04
cg0998		Trypsin-like serine protease	1.0	1.3E−03	3.1	1.1E−03
cg1096		Hypothetical protein	1.0	3.3E−04	1.3	3.6E−02
cg1907		Putative phosphopantothenoylcysteine synthetase/decarboxylase	1.2	1.0E−08	2.8	4.5E−03
cg2030		Hypothetical protein	1.2	2.0E−02	1.2	1.7E−02
cg2092	*sigA*	RNA polymerase sigma factor SigA	3.8	6.4E−11	5.4	3.4E−06
cg2340		ABC-type transporter, substrate-binding protein	1.2	1.0E−03	1.2	1.7E−02
cg2341		Co/Zn/Cd cation efflux transporter	1.0	4.1E−04	1.3	1.2E−03
cg2636	*catA*	Catechol 1,2-dioxygenase	1.2	1.0E−08	1.1	1.2E−02
cg3037	*cls*	Cardiolipin synthase	1.2	1.5E−06	4.6	1.1E−05

a Gene ID, gene name and function of proteins are given according to CoryneRegNet (http://coryneregnet.compbio.sdu.dk/v6/index.html) and from previous studies.

b Relative RNA amount of *sigA* overexpressing strain against the control strain with the empty vector was shown as log 2 values (M-values).

c FDR represents false discovery rate. 50 µM IPTG were added from the beginning of the cultivation.

**Fig. 5:**
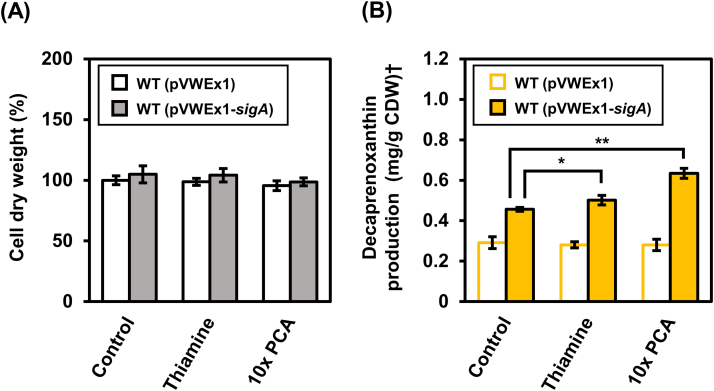
**Effect of thiamine supplementation and increased addition of PCA on biomass formation (A) and decaprenoxanthin production (B).** Cell weights after 48 h of cultivation were determined based on the optical density at 600 nm and normalized to the control cultivation of WT(pVWEx1) with regular CGXII minimal medium (Control). Either 10 μg/L of thiamine (Thiamine) was added or the PCA concentration was raised to 300 mg/L (10xPCA). Decaprenoxanthin production was calculated based on the absorption of carotenoid extracts at wavelength of 440 nm and normalized by cell weight used for extraction. Statistical significance was calculated with paired Student t-test (two-tailed). * and ** represent p-value less than 0.05 and 0.01, respectively. Microscale cultivations were performed in Biolector Flower Plates. Carotenoid production is given as means of biological triplicates with standard deviations. †Decaprenoxanthin production was calculated as β-carotene equivalent.

Based on the finding that *catA* and thiamine biosynthesis genes were induced when *sigA* was overexpressed, growth experiments in the presence of 10-fold increased PCA concentration (300 mg/L) or in the presence of 10 μg/L thiamine were performed. These supplements did not influence the final biomass of *C. glutamicum* WT(pVWEx1) and WT(pVWEx1-*sigA*) (Fig. 5A). Accumulation of decaprenoxanthin by WT(pVWEx1-*sigA*), but not by WT(pVWEx1), increased by about 10% when thiamine was added (Fig. 5B). In the presence of a 10-fold increased PCA concentration, decaprenoxanthin production by WT(pVWEx1-*sigA*), but not by WT(pVWEx1), increased by about 40% (Fig. 5B). Therefore, the finding that thiamine and PCA addition supported decaprenoxanthin production by *C. glutamicum* WT(pVWEx1-*sigA*) is commensurate with the gene expression changes due to *sigA* overexpression.

### 3.6 Deletion of the sigma factor gene *sigB* increased carotenoid production

**Fig. 6:**
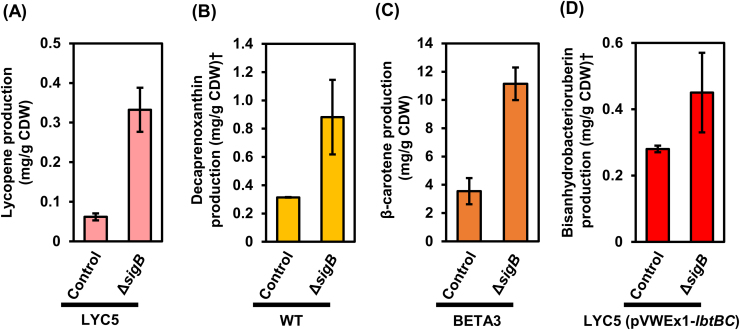
Effect of deletion of *sigB* on lycopene (A), decaprenoxanthin (B), β-carotene (C) and bisanhydrobacterioruberin (D) production by *C. glutamicum*. Lycopene, decaprenoxanthin, β-carotene and bisanhydrobacterioruberin production was calculated 48 h after inoculation based on the absorption of carotenoid extract at respective wavelength, and normalized by cell weight used for extraction. Carotenoid production is given as means of biological triplicates with standard deviations. †Decaprenoxanthin and bisanhydrobacterioruberin production was calculated as β-carotene equivalent.

SigA of *C. glutamicum* is mostly associated to RNA polymerase in the exponential growth phase, while SigB is believed to replace SigA during the transition to the stationary growth phase (Kim et al., 2016, Larisch et al., 2007, Pátek and Nešvera, 2011). During the transition to the stationary growth phase, expression of *sigA* is reduced and expression of *sigB* increased (Larisch et al., 2007). Thus, we hypothesized that overexpression of *sigA* increases the proportion of SigA containing RNA polymerase holoenzymes in the stationary growth phase and that deletion of *sigB* might have a comparable effect. To test this hypothesis, *sigB* was deleted in carotenoid producer strains. The deletion and the control strains grew comparably in glucose minimal medium and consumed glucose within 24 h of cultivation. Extraction and quantification of carotenoids after 48 h of cultivation revealed that about 5-fold more lycopene was produced by LYC5Δ*sigB* in comparison to *C. glutamicum* LYC5 (Fig. 6A), which is comparable to the about 8-fold increased lycopene production as consequence of *sigA* overexpression (Fig. 3). In a similar manner, deletion of *sigB* improved production of decaprenoxanthin, β-carotene and BABR by *C. glutamicum* WT, BETA3 and LYC5(pVWEx1-*lbtBC*), respectively (Fig. 6B–D). Thus, deletion of *sigB* is beneficial for carotenoid production by *C. glutamicum*, likely because a high proportion of SigA containing RNA polymerase holoenzymes can be maintained in the absence of sigma factor competition by SigB. Combination of deletion of *sigB* and overexpression of *sigA* did not further increase lycopene production but perturbed growth (data not shown).

## 4 Discussion

Production of endogenous and non-native carotenoids by *C. glutamicum* was improved by overexpression of the primary sigma factor gene *sigA* and by deletion of the alternative sigma factor gene *sigB*. Overexpression of sigma factor genes has also been used in metabolic engineering of *E. coli* for lycopene production (Alper and Stephanopoulos, 2007, Jin and Stephanopoulos, 2007, Kang et al., 2005). Overexpression of *rpoS,* the general stress sigma factor that regulates expression of hundreds of *E. coli* genes (Weber et al., 2005), increased the lycopene production by recombinant *E. coli* strains (Jin and Stephanopoulos, 2007, Kang et al., 2005). This effect is suggested to be caused by altering cellular oxidative status and preventing degradation of lycopene (Bongers et al., 2015). In an approach called global transcription machinery engineering (gTME), *rpoD*, the gene for the principle sigma factor of *E. coli*, was randomly mutated and overexpressed from a plasmid (Alper and Stephanopoulos, 2007). Libraries of *E. coli* cells with mutated forms of *rpoD* were subjected to selection/screening for ethanol tolerance and lycopene production, and the *rpoD* sequences of the best performing mutants were analyzed (Alper and Stephanopoulos, 2007). Therefore, it is conceivable that the gTME approach may be applied in addition to overexpression of *sigA* for improved carotenoid producing *C. glutamicum* strains.

SigA is considered to be important for transcription of housekeeping genes, especially in the exponential growth phase (Pátek and Nešvera, 2011, Pfeifer-Sancar et al., 2013) and its transcript is more abundant in the exponential phase than in the stationary phase (Larisch et al., 2007). Some of the genes differentially expressed as consequence of *sigA* overexpression (Table 2, S1 and S2) were previously shown to be SigB-dependent (Larisch et al., 2007): cg0096, cg0291, cg0753, cg0899, cg0998, cg1109, cg1147, cg1930, cg2704, cg3022 and cg3330. These genes represent a subset of the 95 SigB-dependent genes (Larisch et al., 2007). In addition, two genes known to be differentially expressed in the stationary phase independent of SigB (Larisch et al., 2007) were affected by *sigA* overexpression: cg1227 and cg2378, two of 16 genes described to belong to this class of genes (Larisch et al., 2007). The finding that *sigA* overexpression affects at least a subset of known SigB-dependent genes is commensurate with the view that SigB replaces SigA in RNA polymerase holoenzymes during the transition from the exponential to the stationary growth phase. Accordingly, deletion of *sigB* increased carotenoid production (Fig. 5) and overexpression of *sigB* decreased lycopene production (Fig. 2). Mutagenesis of *sigB* has been used for metabolic engineering of *C. glutamicum* to improve secretion of green fluorescence protein (GFP) and α-amylase (Watanabe et al., 2012). Deletion of *sigB*, however, is known to impair growth of *C. glutamicum* at acidic pH (Jakob et al., 2007). Secretion of glutathione S-transferase was improved when using a mutated form of the SigB-dependent promoter of cg3141 (Kim et al., 2016). It is not known whether protein secretion or growth at low pH are also influenced by *sigA* overexpression.

Genes directly related to carotenogenesis were neither affected by *sigA* overexpression (Table 2, S1 and S2) nor are they known to be SigB dependent (Larisch et al., 2007). The transcriptional start sites of the carotenoid operons *crtE*-cg0722-*crtBIYeYfEb* (guanosine 114 nucleotides upstream of the first nucleotide of the ATG start codon) and *crtB*2*I*2-1/2 (guanosine thirteen nucleotides upstream of the first nucleotide of the start codon GTG) of *C. glutamicum* have been mapped (Heider et al., 2012). Typical SigA-dependent promoter sequence motifs (Pfeifer-Sancar et al., 2013) were found with the consensus −10 hexamer of *C. glutamicum* promoters (TANNNT) in the −15 to −10 region of *crtE* and a −35 motif of *crtE* sharing four identical nucleotides with the −35 consensus (TTGNCA) hexamer (Heider et al., 2012). These promoters are likely not SigB-dependent since their promoter sequences differ from those of the typical SigB-dependent promoters (−35: GNGNCN; −10: TAMAAT) (Pátek and Nešvera, 2011). Thus, accumulation of carotenoids in the stationary phase has to be due to other/indirect effects. Cardiolipin synthetase is important for lipidogenesis and expression of its gene *cls* was upregulated when *sigA* was overexpressed at 8 h and 24 h (Table 2). Inactivation and overexpression of *cls* have previously been shown to affect the lipid composition of the cellular membrane and temperature sensitivity (Nampoothiri et al., 2002). Thus, it is tempting to speculate that *sigA* overexpression may change the *C. glutamicum* cytoplasmic membrane, which in turn may be beneficial for carotenoid accumulation. However, it remains to be shown if modification of the lipid composition of the cytoplasmic membrane influences carotenoid accumulation. Genes of carbon metabolism were differentially expressed when *sigA* was overexpressed. The genes for the glycolytic enzyme phosphofructokinase *pfkA* and for pyruvate quinone dehydrogenase *pqo* were shown to be upregulated during the transition and in the stationary phase (Ehira et al., 2008), which is downregulated at 24 h due to *sigA* overexpression in this work (Table S2). This argues against increased availability of the glycolytic intermediates pyruvate and glyceraldehyde 3-phosphate for IPP synthesis as consequence of *sigA* overexpression.

The catechol 1,2-diogxygenase gene *catA* was upregulated both at 8 h and 24 h when *sigA* was overexpressed (Table 2). CatA and the dioxygenase required for PCA degradation are iron containing enzymes. At a 10-fold higher concentration of the iron chelator PCA, more carotenoid was produced (Fig. 4). Transcription of *catA* is directly repressed by iron homeostasis regulator RipA (Wennerhold et al., 2005). Besides *catA*, other members of the RipA regulon (Wennerhold and Bott, 2006), namely *acn* encoding the citric acid cycle iron sulfur cluster-containing enzyme aconitase*,* the heme biosynthesis gene *hemH* and *ftn* coding for an iron storage protein showed differential gene expression as consequence of *sigA* overexpression (increased RNA levels of *acn* and *hemH*, decreased RNA levels for *ftn*; Appendix A, Appendix A). Iron availability is expected to influence carotenogenesis since two enzymes of the MEP pathway are iron-sulfur proteins: IspG (Lee et al., 2010) and IspH (Gräwert et al., 2004). However, it has to be noted that genes of iron homeostasis tend to change expression levels in growth experiments with CGXII minimal medium due to the limited availability of iron and the iron chelator PCA (Krug et al., 2005, Liebl et al., 1989, Merkens et al., 2005, Unthan et al., 2014).

Carotenogenesis, pyridoxal (vitamin B6) biosynthesis (Hill et al., 1989) and thiamine biosynthesis (Begley et al., 1999) are interrelated as the condensation of glyceraldehyde 3-phosphate and pyruvate to 1-deoxyxylulose-5-phosphate (DXP) catalyzed by Dxs is the first step of the IPP, pyridoxal and thiamine biosynthetic pathways. Interestingly, expression of pyridoxal 5′-phosphate synthase genes (*pdxST*) was upregulated at 8 h as consequence of *sigA* overexpression (Table S1). Dxs requires thiamine pyrophosphate as cofactor (Sprenger et al., 1997) and is considered to be one of the rate-limiting steps of the MEP pathway (Estévez et al., 2001) as has also been shown for *C. glutamicum* (Heider et al., 2014c). Some genes of thiamine synthesis were shown to be upregulated at 8 h or 24 h upon *sigA* overexpression. Thiamine supplementation has been shown to be beneficial for carotenoid production were reported in *Corynebacterium* species, for example, *Corynebacterium poinsettiae* (Starr and Saperstein, 1953) and *Corynebacterium michiganense* (Saperstein et al., 1954). As shown here, thiamine was only limiting for carotenoid production when *sigA* was overexpressed (Fig. 4).

## 5 Conclusion

Taken together, overexpression of the gene for the primary sigma factor SigA in *C. glutamicum* led to pleiotropic effects, as expected. It has to be noted that it may be difficult to transfer this approach to other production strains and that it may be necessary to repeat screens of sigma factor gene overexpression for each production process. The beneficial effect of *sigA* overexpression on carotenoid production may be due to these pleiotropic effects involving direct metabolic links to thiamine and pyridoxal biosynthesis (DXP as common biosynthetic precursor) and indirect effects on cardiolipin biosynthesis (cytoplasmic membrane composition) as well as iron availability (iron sulfur cluster-containing enzymes IspG and IspH in the MEP pathway). These pleiotropic effects were shown to be beneficial for overproduction of lycopene, the endogenous decaprenoxanthin and the non-native carotenoids bisanhydrobacterioruberin and β-carotene.

## References

[bib1] Abbes M., Baati H., Guermazi S., Messina C., Santulli A., Gharsallah N., Ammar E. (2013). Biological properties of carotenoids extracted from *Halobacterium halobium* isolated from a Tunisian solar saltern. BMC Complement. Altern. Med..

[bib2] Alper H., Stephanopoulos G. (2007). Global transcription machinery engineering: a new approach for improving cellular phenotype. Metab. Eng..

[bib3] Ausich R.L. (2009). Commercial opportunities for carotenoid production by biotechnology. Pure Appl. Chem..

[bib4] Begley T.P., Downs D.M., Ealick S.E., McLafferty F.W., Van Loon A.P., Taylor S., Campobasso N., Chiu H.J., Kinsland C., Reddick J.J., Xi J. (1999). Thiamin biosynthesis in prokaryotes. Arch. Microbiol..

[bib5] Bongers M., Chrysanthopoulos P.K., Behrendorff J.B.Y.H., Hodson M.P., Vickers C.E., Nielsen L.K. (2015). Systems analysis of methylerythritol-phosphate pathway flux in *E. coli*: insights into the role of oxidative stress and the validity of lycopene as an isoprenoid reporter metabolite. Microb. Cell Factor..

[bib6] Breithaupt, D.E., 2007. Modern application of xanthophylls in animal feeding – a review. In: Trends Food Sci. Technol. 4th International Congress on Pigments in Food, Vol. 18, pp. 501–506. 〈http://doi.org/10.1016/j.tifs.2007.04.009〉.

[bib7] Britton, G., Liaaen-Jensen, S., Pfander, H., 2004. Carotenoids. Birkhäuser Basel, Basel, Switzerland.

[bib8] Chang W., Song H., Liu H., Liu P. (2013). Current development in isoprenoid precursor biosynthesis and regulation. Curr. Opin. Chem. Biol..

[bib9] Dondrup M., Albaum S.P., Griebel T., Henckel K., Jünemann S., Kahlke T., Kleindt C.K., Küster H., Linke B., Mertens D., Mittard-Runte V., Neuweger H., Runte K.J., Tauch A., Tille F., Pühler A., Goesmann A. (2009). EMMA 2 – a MAGE-compliant system for the collaborative analysis and integration of microarray data. BMC Bioinform..

[bib10] Eggeling L., Bott M. (2005). Handbook of *Corynebacterium glutamicum*.

[bib11] Ehira S., Shirai T., Teramoto H., Inui M., Yukawa H. (2008). Group 2 sigma factor SigB of *Corynebacterium glutamicum* positively regulates glucose metabolism under conditions of oxygen deprivation. Appl. Environ. Microbiol..

[bib12] Estévez J.M., Cantero A., Reindl A., Reichler S., León P. (2001). 1-Deoxy-D-xylulose-5-phosphate synthase, a limiting enzyme for plastidic isoprenoid biosynthesis in plants. J. Biol. Chem..

[bib13] Feklístov A., Sharon B.D., Darst S.A., Gross C.A. (2014). Bacterial sigma factors: a historical, structural, and genomic perspective. Annu. Rev. Microbiol..

[bib14] Gibson D.G., Young L., Chuang R.-Y., Venter J.C., Hutchison C.A., Smith H.O. (2009). Enzymatic assembly of DNA molecules up to several hundred kilobases. Nat. Methods.

[bib15] Gräwert T., Kaiser J., Zepeck F., Laupitz R., Hecht S., Amslinger S., Schramek N., Schleicher E., Weber S., Haslbeck M., Buchner J., Rieder C., Arigoni D., Bacher A., Eisenreich W., Rohdich F. (2004). IspH protein of *Escherichia coli*: studies on iron-sulfur cluster implementation and catalysis. J. Am. Chem. Soc..

[bib16] Gruber T.M., Gross C.A. (2003). Multiple sigma subunits and the partitioning of bacterial transcription space. Annu. Rev. Microbiol..

[bib17] Heider S.A.E., Peters-Wendisch P., Netzer R., Stafnes M., Brautaset T., Wendisch V.F. (2014). Production and glucosylation of C50 and C40 carotenoids by metabolically engineered *Corynebacterium glutamicum*. Appl. Microbiol. Biotechnol..

[bib18] Heider S.A.E., Peters-Wendisch P., Wendisch V.F. (2012). Carotenoid biosynthesis and overproduction in *Corynebacterium glutamicum*. BMC Microbiol..

[bib19] Heider S.A.E., Peters-Wendisch P., Wendisch V.F., Beekwilder J., Brautaset T. (2014). Metabolic engineering for the microbial production of carotenoids and related products with a focus on the rare C50 carotenoids. Appl. Microbiol. Biotechnol..

[bib20] Heider S.A.E., Wolf N., Hofemeier A., Peters-Wendisch P., Wendisch V.F. (2014). Optimization of the IPP precursor supply for the production of lycopene, Decaprenoxanthin and Astaxanthin by *Corynebacterium glutamicum*. Front. Bioeng. Biotechnol..

[bib21] Henke N.A., Heider S.A.E., Peters-Wendisch P., Wendisch V.F. (2016). Production of the marine carotenoid Astaxanthin by Metabolically engineered *Corynebacterium glutamicum*. Mar. Drugs.

[bib22] Hill R.E., Sayer B.G., Spenser I.D. (1989). Biosynthesis of vitamin B6: incorporation of D-1-deoxyxylulose. J. Am. Chem. Soc..

[bib23] Jakob K., Satorhelyi P., Lange C., Wendisch V.F., Silakowski B., Scherer S., Neuhaus K. (2007). Gene expression analysis of *Corynebacterium glutamicum* subjected to long-term lactic acid adaptation. J. Bacteriol..

[bib24] Jin Y.-S., Stephanopoulos G. (2007). Multi-dimensional gene target search for improving lycopene biosynthesis in *Escherichia coli*. Metab. Eng..

[bib25] Kang M.J., Lee Y.M., Yoon S.H., Kim J.H., Ock S.W., Jung K.H., Shin Y.C., Keasling J.D., Kim S.W. (2005). Identification of genes affecting lycopene accumulation in *Escherichia coli* using a shot-gun method. Biotechnol. Bioeng..

[bib26] Kim M.J., Yim S.S., Choi J.W., Jeong K.J. (2016). Development of a potential stationary-phase specific gene expression system by engineering of SigB-dependent cg3141 promoter in *Corynebacterium glutamicum*. Appl. Microbiol. Biotechnol..

[bib27] Knoll A., Bartsch S., Husemann B., Engel P., Schroer K., Ribeiro B., Stöckmann C., Seletzky J., Büchs J. (2007). High cell density cultivation of recombinant yeasts and bacteria under non-pressurized and pressurized conditions in stirred tank bioreactors. J. Biotechnol..

[bib28] Krubasik P., Takaichi S., Maoka T., Kobayashi M., Masamoto K., Sandmann G. (2001). Detailed biosynthetic pathway to decaprenoxanthin diglucoside in *Corynebacterium glutamicum* and identification of novel intermediates. Arch. Microbiol..

[bib29] Krug A., Wendisch V.F., Bott M. (2005). Identification of AcnR, a TetR-type repressor of the aconitase gene *acn* in *Corynebacterium glutamicum*. J. Biol. Chem..

[bib30] Larisch C., Nakunst D., Hüser A.T., Tauch A., Kalinowski J. (2007). The alternative sigma factor SigB of *Corynebacterium glutamicum* modulates global gene expression during transition from exponential growth to stationary phase. BMC Genom..

[bib31] Lazrak T., Milon A., Wolff G., Albrecht A.M., Miehé M., Ourisson G., Nakatani Y. (1987). Comparison of the effects of inserted C40- and C50-terminally dihydroxylated carotenoids on the mechanical properties of various phospholipid vesicles. Biochim. Biophys. Acta.

[bib32] Lee M., Gräwert T., Quitterer F., Rohdich F., Eppinger J., Eisenreich W., Bacher A., Groll M. (2010). Biosynthesis of isoprenoids: crystal structure of the [4Fe-4S] cluster protein IspG. J. Mol. Biol..

[bib33] Liebl W., Bayerl A., Schein B., Stillner U., Schleifer K.H. (1989). High efficiency electroporation of intact *Corynebacterium glutamicum* cells. FEMS Microbiol. Lett..

[bib34] Merkens H., Beckers G., Wirtz A., Burkovski A. (2005). Vanillate metabolism in *Corynebacterium glutamicum*. Curr. Microbiol..

[bib35] Misawa N., Shimada H. (1997). Metabolic engineering for the production of carotenoids in non-carotenogenic bacteria and yeasts. J. Biotechnol..

[bib36] Moise A.R., Al-Babili S., Wurtzel E.T. (2014). Mechanistic aspects of carotenoid biosynthesis. Chem. Rev..

[bib37] Nampoothiri K.M., Hoischen C., Bathe B., Möckel B., Pfefferle W., Krumbach K., Sahm H., Eggeling L. (2002). Expression of genes of lipid synthesis and altered lipid composition modulates L-glutamate efflux of *Corynebacterium glutamicum*. Appl. Microbiol. Biotechnol..

[bib38] Netzer R., Krause M., Rittmann D., Peters-Wendisch P.G., Eggeling L., Wendisch V.F., Sahm H. (2004). Roles of pyruvate kinase and malic enzyme in *Corynebacterium glutamicum* for growth on carbon sources requiring gluconeogenesis. Arch. Microbiol..

[bib39] Netzer R., Stafsnes M.H., Andreassen T., Goksøyr A., Bruheim P., Brautaset T. (2010). Biosynthetic pathway for γ-cyclic sarcinaxanthin in *Micrococcus luteus*: heterologous expression and evidence for diverse and multiple catalytic functions of C(50) carotenoid cyclases. J. Bacteriol..

[bib40] Norgård S., Aasen A.J., Liaaen-Jensen S. (1970). Bacterial carotenoids. 32. C50-carotenoids 6. Carotenoids from *Corynebacterium poinsettiae* including four new C50-diols. Acta Chem. Scand..

[bib41] Osanai T., Numata K., Oikawa A., Kuwahara A., Iijima H., Doi Y., Tanaka K., Saito K., Hirai M.Y. (2013). Increased bioplastic production with an RNA polymerase sigma factor SigE during nitrogen starvation in *Synechocystis* sp. PCC 6803. DNA Res. Int. J. Rapid Publ. Rep. Genes Genomes.

[bib42] Pátek M., Nešvera J. (2011). Sigma factors and promoters in *Corynebacterium glutamicum*. J. Biotechnol..

[bib43] Peters-Wendisch P.G., Schiel B., Wendisch V.F., Katsoulidis E., Möckel B., Sahm H., Eikmanns B.J. (2001). Pyruvate carboxylase is a major bottleneck for glutamate and lysine production by *Corynebacterium glutamicum*. J. Mol. Microbiol. Biotechnol..

[bib44] Pfeifer-Sancar K., Mentz A., Rückert C., Kalinowski J. (2013). Comprehensive analysis of the *Corynebacterium glutamicum* transcriptome using an improved RNAseq technique. BMC Genom..

[bib45] Polen T., Schluesener D., Poetsch A., Bott M., Wendisch V.F. (2007). Characterization of citrate utilization in *Corynebacterium glutamicum* by transcriptome and proteome analysis. FEMS Microbiol. Lett..

[bib46] Qian Z.-G., Xia X.-X., Lee S.Y. (2009). Metabolic engineering of *Escherichia coli* for the production of putrescine: a four carbon diamine. Biotechnol. Bioeng..

[bib47] Sambrook J. (2001). Molecular Cloning: A Laboratory Manual.

[bib48] Saperstein S., Starr M.P., Filfus J.A. (1954). Alterations in carotenoid synthesis accompanying mutation in *Corynebacterium michiganense*. J. Gen. Microbiol..

[bib49] Schäfer A., Tauch A., Jäger W., Kalinowski J., Thierbach G., Pühler A. (1994). Small mobilizable multi-purpose cloning vectors derived from the *Escherichia coli* plasmids pK18 and pK19: selection of defined deletions in the chromosome of *Corynebacterium glutamicum*. Gene.

[bib50] Scotter M.J. (2011). Methods for the determination of European Union-permitted added natural colours in foods: a review. Food Addit. Contam. Part Chem. Anal. Control Expo. Risk Assess..

[bib51] Shen X.-H., Liu Z.-P., Liu S.-J. (2004). Functional identification of the gene locus (ncg12319 and characterization of catechol 1,2-dioxygenase in Corynebacterium glutamicum. Biotechnol. Lett..

[bib52] Sprenger G.A., Schörken U., Wiegert T., Grolle S., de Graaf A.A., Taylor S.V., Begley T.P., Bringer-Meyer S., Sahm H. (1997). Identification of a thiamin-dependent synthase in *Escherichia coli* required for the formation of the 1-deoxy-D-xylulose 5-phosphate precursor to isoprenoids, thiamin, and pyridoxol. Proc. Natl. Acad. Sci. USA.

[bib53] Stansen C., Uy D., Delaunay S., Eggeling L., Goergen J.-L., Wendisch V.F. (2005). Characterization of a *Corynebacterium glutamicum* lactate utilization operon induced during temperature-triggered glutamate production. Appl. Environ. Microbiol..

[bib54] Starr M.P., Saperstein S. (1953). Thiamine and the carotenoid pigments of *Corynebacterium poinsettiae*. Arch. Biochem. Biophys..

[bib55] Stevens D.C., Conway K.R., Pearce N., Villegas-Peñaranda L.R., Garza A.G., Boddy C.N. (2013). Alternative sigma factor Over-expression enables heterologous expression of a type II polyketide biosynthetic pathway in *Escherichia coli*. PLoS One.

[bib56] Taniguchi H., Wendisch V.F. (2015). Exploring the role of sigma factor gene expression on production by *Corynebacterium glutamicum*: sigma factor H and FMN as example. Front. Microbiol..

[bib57] Tao L., Yao H., Cheng Q. (2007). Genes from a *Dietzia* sp. for synthesis of C40 and C50 beta-cyclic carotenoids. Gene.

[bib58] Toyoda K., Inui M. (2016). Regulons of global transcription factors in *Corynebacterium glutamicum*. Appl. Microbiol. Biotechnol..

[bib59] Tripathi L., Zhang Y., Lin Z. (2014). Bacterial sigma factors as targets for engineered or synthetic transcriptional control. Front. Bioeng. Biotechnol..

[bib60] Unthan S., Grünberger A., van Ooyen J., Gätgens J., Heinrich J., Paczia N., Wiechert W., Kohlheyer D., Noack S. (2014). Beyond growth rate 0.6: What drives *Corynebacterium glutamicum* to higher growth rates in defined medium. Biotechnol. Bioeng..

[bib61] van der Rest M.E., Lange C., Molenaar D. (1999). A heat shock following electroporation induces highly efficient transformation of *Corynebacterium glutamicum* with xenogeneic plasmid DNA. Appl. Microbiol. Biotechnol..

[bib62] Vranová E., Coman D., Gruissem W. (2013). Network analysis of the MVA and MEP pathways for isoprenoid synthesis. Annu. Rev. Plant Biol..

[bib63] Watanabe K., Teramoto H., Suzuki N., Inui M., Yukawa H. (2012). Influence of SigB inactivation on *Corynebacterium glutamicum* protein secretion. Appl. Microbiol. Biotechnol..

[bib64] Weber H., Polen T., Heuveling J., Wendisch V.F., Hengge R. (2005). Genome-wide analysis of the general stress response network in *Escherichia coli*: sigmaS-dependent genes, promoters, and sigma factor selectivity. J. Bacteriol..

[bib65] Wendisch V.F. (2003). Genome-wide expression analysis in Corynebacterium glutamicum using DNA microarrays. J. Biotechnol..

[bib66] Wendisch V.F. (2014). Microbial production of amino acids and derived chemicals: synthetic biology approaches to strain development. Curr. Opin. Biotechnol..

[bib67] Wennerhold J., Bott M. (2006). The DtxR regulon of *Corynebacterium glutamicum*. J. Bacteriol..

[bib68] Wennerhold J., Krug A., Bott M. (2005). The AraC-type regulator RipA represses aconitase and other iron proteins from *Corynebacterium* under iron limitation and is itself repressed by DtxR. J. Biol. Chem..

[bib69] Ye V.M., Bhatia S.K. (2012). Pathway engineering strategies for production of beneficial carotenoids in microbial hosts. Biotechnol. Lett..

