# Supplementary material for: Overexpression of the primary sigma factor gene sigA improved carotenoid production by Corynebacterium glutamicum: Application to production of β-carotene and the non-native linear C50 carotenoid bisanhydrobacterioruberin
Source: Metab Eng Commun. 2017 Jan 13;4:1–11. doi: 10.1016/j.meteno.2017.01.001 (PMC5678898; doi:10.1016/j.meteno.2017.01.001)
Supplement: Table S1 — Supplementary material [file mmc1.docx]

**Supplementary material**

**To**

**Overexpression of the primary sigma factor gene *sigA* improved carotenoid production by *Corynebacterium glutamicum*: application to production of β-carotene and the non-native linear C50 carotenoid bisanhydrobacterioruberin**

Hironori Taniguchi, Nadja A. Henke, Sabine A. E. Heider & Volker F. Wendisch^#^

Chair of Genetics of Prokaryotes, Faculty of Biology & CeBiTec, Bielefeld University, Bielefeld, Germany;

^#^Corresponding author: phone: +49-521-106 5611; volker.wendisch@uni-bielefeld.de

Table S1. DNA microarray analysis of genes differentially expressed upon *sigA* overexpression after 8 hours of cultivation

| 50 genes | | **Up-regulated genes** | |  | |
| --- | --- | --- | --- | --- | --- |
| gene ID^a^ | name^a^ | Function of protein^a^ | M-value^b^ | | FDR^c^ |
| cg0096 |  | Conserved hypothetical protein | 1.1 | | 3.5E-04 |
| cg0107 |  | Putative secreted protein | 1.0 | | 2.0E-06 |
| cg0291 |  | Putative dioxygenase | 1.1 | | 1.7E-03 |
| cg0808 | *wbpC* | Conserved putative membrane protein | 1.2 | | 1.5E-05 |
| cg0898 | *pdxS* | pyridoxal 5'-phosphate (PLP) synthase subunit | 1.4 | | 2.3E-06 |
| cg0899 | *pdxT* | pyridoxal 5'-phosphate (PLP) synthase subunit, glutamine amidotransferase | 1.0 | | 4.8E-04 |
| cg0998 |  | Trypsin-like serine protease | 1.0 | | 1.3E-03 |
| cg1088 |  | ABC-type putative multidrug transporter, ATPase and permease subunit | 1.2 | | 2.3E-05 |
| cg1095 |  | Hypothetical protein | 1.4 | | 1.2E-04 |
| cg1096 |  | Hypothetical protein | 1.0 | | 3.3E-04 |
| cg1109 |  | Hypothetical protein | 1.1 | | 4.9E-06 |
| cg1139 |  | Allophanate hydrolase subunit 2 | 1.1 | | 6.6E-05 |
| cg1140 |  | Allophanate hydrolase subunit 1 | 1.0 | | 1.2E-05 |
| cg1142 |  | Putative Mn2+ transporter, metal ion (Mn2+-iron) transporter (Nramp) family | 1.3 | | 1.9E-06 |
| cg1147 | *ssuI* | NAD(P)H-dependent FMN reductase | 1.2 | | 5.2E-03 |
| cg1210 |  | Putative membrane protein | 1.4 | | 3.4E-05 |
| cg1227 |  | Putative membrane protein | 1.4 | | 1.6E-08 |
| cg1230 |  | Conserved hypothetical protein | 1.1 | | 4.9E-06 |
| cg1231 | *chaA* | Putative secondary Na+/Ca2+ antiporter, Ca2+:cation antiporter (CaCA) Family | 1.1 | | 1.1E-05 |
| cg1232 |  | Conserved hypothetical protein, LmbE-family | 1.2 | | 3.4E-03 |
| cg1294 |  | Putative esterase, alpha-beta hydrolase superfamily | 1.0 | | 7.4E-05 |
| cg1391 |  | Conserved hypothetical protein, related to capsule biosynthesis enzymes | 1.3 | | 7.0E-07 |
| cg1513 | *tnp23a(ISCg23a)* | Transposase, putative pseudogene | 1.1 | | 8.9E-07 |
| cg1552 | *qorR* | transcriptional repressor of quinone oxidoreductase qor2 | 1.0 | | 9.1E-03 |
| cg1902 |  | Putative secreted protein | 1.1 | | 1.8E-03 |
| cg1907 |  | Putative phosphopantothenoylcysteine synthetase/decarboxylase | 1.2 | | 1.0E-08 |
| cg1908 |  | Hypothetical protein | 1.5 | | 1.0E-06 |
| cg1909 |  | Hypothetical protein | 2.3 | | 4.0E-06 |
| cg2030 |  | Hypothetical protein | 1.2 | | 2.0E-02 |
| cg2036 |  | Putative secreted protein | 1.4 | | 5.3E-04 |
| cg2046 |  | Hypothetical protein | 1.6 | | 5.2E-05 |
| cg2053 |  | Putative membrane protein | 1.5 | | 4.9E-06 |
| cg2070 | *'int2* | Putative phage integrase (C-terminal fragment) | 1.2 | | 8.6E-06 |
| cg2092 | *sigA* | RNA polymerase sigma factor rpoD (Sigma-A) | 3.8 | | 6.4E-11 |
| cg2237 | *thiO* | Putative D-amino acid dehydrogenase, small subunit | 1.2 | | 6.6E-05 |
| cg2238 | *thiS* | Sulfur transfer protein involved in thiamine biosynthesis, ThiS-like | 1.2 | | 1.4E-06 |
| cg2239 | *thiG* | Thiamine biosynthesis protein, ThiG-like | 1.1 | | 2.2E-09 |
| cg2340 |  | ABC-type putative amino acid transporter, substrate-binding lipoprotein | 1.2 | | 1.0E-03 |
| cg2341 |  | Putative Co/Zn/Cd cation transporter | 1.0 | | 4.1E-04 |
| cg2438 |  | Hypothetical protein | 1.7 | | 7.0E-06 |
| cg2636 | *catA* | Catechol 1,2-dioxygenase | 1.2 | | 1.0E-08 |
| cg2836 | *sucD* | Succinate--CoA ligase (ADP-forming), alpha subunit | 1.0 | | 6.1E-08 |
| cg3037 | *cls* | Cardiolipin synthase | 1.2 | | 1.5E-06 |
| cg3269 |  | Putative membrane protein, putative pseudogene | 1.1 | | 7.8E-04 |
| cg3270 |  | Putative membrane protein, putative pseudogene (N-terminal fragment) | 1.1 | | 3.9E-04 |
| cg3280 |  | Putative secreted protein | 1.2 | | 5.1E-04 |
| cg3330 |  | Putative secreted protein | 1.3 | | 3.6E-03 |
| cg3368 |  | ABC-type putative multidrug transporter, permease subunit | 1.1 | | 8.1E-04 |
| cg3408 |  | Hypothetical protein | 1.1 | | 1.5E-03 |
| cg4006 |  | Hypothetical protein | 1.3 | | 1.4E-02 |
|  | |  | | | |
| 18 genes | | ***Down regulated genes*** | |  | |
| gene ID^a^ | gene  name^a^ | Function of protein^a^ | M-value^b^ | | FDR^c^ |
| cg0091 |  | Putative D-isomer specific 2-hydroxyacid dehydrogenase | -1.1 | | 4.5E-05 |
| cg0131 |  | Putative aldo-keto reductase | -1.2 | | 6.9E-05 |
| cg0195 |  | Putative membrane protein | -1.1 | | 5.8E-04 |
| cg0612 | *dkg* | Putative aldo/keto reductase, related to diketogulonate reductase | -1.2 | | 5.6E-06 |
| cg0828 |  | Putative dihydrofolate reductase | -1.0 | | 1.3E-04 |
| cg0975 |  | Putative chorismate mutase | -1.2 | | 9.4E-05 |
| cg1068 |  | Putative oxidoreductase | -1.1 | | 2.6E-04 |
| cg1418 |  | ABC-type putative iron-siderophore transporter, substrate-binding lipoprotein | -1.0 | | 5.4E-03 |
| cg1702 |  | Hypothetical protein | -1.2 | | 1.6E-05 |
| cg1918 |  | Putative secreted protein | -1.1 | | 6.4E-06 |
| cg2156 |  | Hypothetical protein | -1.0 | | 3.9E-03 |
| cg2478 |  | Putative penicillin binding protein | -1.0 | | 1.6E-02 |
| cg2546 |  | Putative secondary C4-dicarboxylate transporter, tripartite ATP-independent transporter (TRAP-T) family | -1.0 | | 5.4E-03 |
| cg2838 |  | Putative dithiol-disulfide isomerase | -2.2 | | 9.4E-07 |
| cg3107 | *adhA* | Alcohol dehydrogenase | -1.0 | | 2.5E-05 |
| cg3129 |  | ABC-type transporter, ATPase subunit | -1.6 | | 5.4E-05 |
| cg3155 | *dcd* | dCTP deaminase | -1.0 | | 5.2E-06 |
| cg3405 |  | NADPH:quinone reductase Zn-dependent oxidoreductase | -1.1 | | 1.4E-02 |

^a^ Gene ID, gene name and function of proteins are given according to CoryneRegNet (http://coryneregnet.compbio.sdu.dk/v6/index.html) and from previous studies. ^b^ Relative RNA amount of *sigA* overexpressing strain against the control strain with the empty vector was shown as log 2 values (M-values). ^c^ FDR represents false discovery rate. 50 µM IPTG were added from the beginning of the cultivation.

Table S2. DNA microarray analysis of genes differentially expressed upon *sigA* overexpression after 24 hours of cultivation

| 65 genes | | Up-regulated genes |  |  | |  |
| --- | --- | --- | --- | --- | --- | --- |
| gene ID^a^ | gene  name^a^ | Function of protein^a^ | M-value^b^ | FDR^c^ | |  |
| cg0171 |  | Putative secreted protein | 1.5 | 2.9E-03 | |  |
| cg0311 |  | Putative secreted protein | 1.2 | 3.6E-04 | |  |
| cg0413 | *cmt1* | Trehalose corynomycolyl transferase | 1.4 | 1.4E-02 | |  |
| cg0421 | *wzx* | Putative PST O-antigen protein, multidrug/oligosaccharidyl-lipid/polysaccharide (MOP) translocase | 1.1 | 2.3E-03 | |  |
| cg0422 | *murA* | UDP-N-acetylglucosamine 1-carboxyvinyltransferase | 1.3 | 2.9E-03 | |  |
| cg0495 |  | Putative phosphatase | 2.9 | 3.7E-05 | |  |
| cg0659 |  | Putative acetyltransferase, GNAT-family | 1.7 | 2.7E-03 | |  |
| cg0683 |  | Putative permease | 1.0 | 6.6E-03 | |  |
| cg0690 | *groES* | 10kDa chaperonin | 3.0 | 4.0E-05 | |  |
| cg0693 | *'groEL* | 60kDa chaperonin, putative pseudogene (C-terminal fragment) | 2.9 | 2.3E-03 | |  |
| cg0753 |  | Putative secreted protein | 1.6 | 2.4E-02 | |  |
| cg0998 |  | Trypsin-like serine protease | 3.1 | 1.1E-03 | |  |
| cg1070 |  | Conserved hypothetical protein | 1.8 | 1.2E-03 | |  |
| cg1096 |  | Hypothetical protein | 1.3 | 3.6E-02 | |  |
| cg1121 |  | Permease, MFS-type | 1.0 | 2.6E-02 | |  |
| cg1271 | *sigE* | RNA polymerase sigma factor, ECF-family | 2.0 | 2.2E-05 | |  |
| cg1277 |  | Conserved putative membrane protein | 1.2 | 1.4E-02 | |  |
| cg1334 | *lysA* | Diaminopimelate decarboxylase | 5.9 | 2.7E-08 | |  |
| cg1383 |  | ABC-type putative molybdenum transporter, ATPase subunit | 1.0 | 5.2E-03 | |  |
| cg1466 |  | Putative secreted protein | 1.1 | 3.2E-03 | |  |
| cg1476 | *thiC* | Thiamine biosynthesis protein ThiC | 1.7 | 3.4E-02 | |  |
| cg1514 |  | Secreted protein | 1.2 | 1.7E-06 | |  |
| cg1654 | *thiD1* | Phosphomethylpyrimidine kinase/thiamine-phosphate diphosphorylase | 1.7 | 1.6E-03 | |  |
| cg1685 | *tatX* | Putative twin arginine targeting (Tat) Preprotein translocase subunit | 1.2 | 6.3E-04 | |  |
| cg1734 | *hemH* | Ferrochelatase | 1.3 | 1.9E-02 | |  |
| cg1737 | *acn* | Aconitate hydratase | 1.5 | 1.1E-03 | |  |
| cg1821 |  | Conserved hypothetical protein | 1.7 | 2.5E-03 | |  |
| cg1859 |  | Putative secreted protein | 2.1 | 1.9E-03 | |  |
| cg1903 |  | ABC-type transporter, ATPase subunit | 2.1 | 1.0E-03 | |  |
| cg1904 |  | ABC-type transporter, permease subunit | 1.4 | 4.0E-03 | |  |
| cg1905 |  | Hypothetical protein | 2.5 | 8.2E-04 | |  |
| cg1907 |  | Putative phosphopantothenoylcysteine synthetase/decarboxylase | 2.8 | 4.5E-03 | |  |
| cg1930 |  | Putative secreted hydrolase | 2.1 | 1.0E-04 | |  |
| cg1931 |  | Putative secreted protein | 1.8 | 6.9E-05 | |  |
| cg1966 |  | Hypothetical protein | 1.2 | 2.1E-04 | |  |
| cg2030 |  | Hypothetical protein | 1.2 | 1.7E-02 | |  |
| cg2034 |  | Hypothetical protein | 1.3 | 3.8E-03 | |  |
| cg2037 |  | Conserved hypothetical protein | 1.2 | 1.7E-04 | |  |
| cg2040 |  | Putative transcriptional regulator, HTH_3-family | 1.5 | 6.5E-03 | |  |
| cg2052 |  | Putative secreted protein | 1.1 | 4.6E-02 | |  |
| cg2071 | *int2'* | Putative phage Integrase (N-terminal fragment) | 1.6 | 2.1E-02 | |  |
| cg2092 | *sigA* | RNA polymerase sigma factor *rpoD* (Sigma-A) | 5.4 | 3.4E-06 | |  |
| cg2114 | *lexA* | Putative transcriptional regulator, LexA-family | 1.3 | 1.4E-03 | |  |
| cg2280 | *gdh* | Glutamate dehydrogenase (NADP(+)) | 2.1 | 2.5E-03 | |  |
| cg2306 |  | Conserved hypothetical protein | 1.6 | 3.7E-03 | |  |
| cg2308 |  | Putative secreted protein | 2.7 | 4.6E-02 | |  |
| cg2339 |  | Putative secondary chloramphenicol transporter, drug/metabolite transporter (DMT) superfamily | 1.6 | 1.8E-02 | |  |
| cg2340 |  | ABC-type putative amino acid transporter, substrate-binding lipoprotein | 1.2 | 1.7E-02 | |  |
| cg2341 |  | Putative Co/Zn/Cd cation transporter | 1.3 | 1.2E-03 | |  |
| cg2378 | *mraZ* | Putative MraZ protein | 1.5 | 4.4E-03 | |  |
| cg2565 |  | Hypothetical protein | 1.1 | 4.9E-02 | |  |
| cg2572 |  | Conserved hypothetical protein | 1.2 | 2.3E-06 | |  |
| cg2617 | *vanB* | Vanillate O-demethylase oxidoreductase | 5.2 | 2.7E-05 | |  |
| cg2636 | *catA* | Catechol 1,2-dioxygenase | 1.1 | 1.2E-02 | |  |
| cg2704 |  | ABC-type putative sugar transporter, permease subunit | 2.7 | 1.9E-03 | |  |
| cg2707 |  | Conserved hypothetical protein | 1.0 | 2.3E-03 | |  |
| cg3011 | *groEL* | Chaperonin Cpn60 (60Kd subunit) | 3.2 | 5.4E-05 | |  |
| cg3022 |  | Conserved hypothetical protein | 1.3 | 3.8E-05 | |  |
| cg3037 | *cls* | Cardiolipin synthase | 4.6 | 1.1E-05 | |  |
| cg3225 |  | Putative serine/threonine-specific protein phosphatase | 1.9 | 9.2E-05 | |  |
| cg3282 |  | Putative Cu2+ transporting P-type ATPase | 1.2 | 2.7E-02 | |  |
| cg3283 |  | Hypothetical protein | 1.7 | 9.6E-07 | |  |
| cg3335 | *malE* | Malate dehydrogenase (oxaloacetate-decarboxylating) (NADP(+)) | 1.2 | 5.0E-02 | |  |
| cg3367 |  | ABC-type putative multidrug transporter, ATPase subunit | 1.2 | 2.9E-03 | |  |
| cg4005 |  | Putative secreted protein | 2.1 | 1.4E-02 | |  |
|  | |  |  |  | |  |
| 32 genes | | **Down regulated genes** |  |  | |  |
| gene ID^a^ | gene  name^a^ | Function of protein^a^ | M-value^b^ | FDR^c^ | |  |
| cg0047 |  | Conserved hypothetical protein | -1.6 | 8.8E-05 | |  |
| cg0120 |  | Putative hydrolase | -1.8 | 1.4E-04 | |  |
| cg0129 | *putA* | Proline dehydrogenase/delta-1-pyrroline-5-carboxylate dehydrogenase | -1.7 | 2.5E-05 | |  |
| cg0242 |  | Hypothetical protein | -1.9 | 4.4E-06 | |  |
| cg0260 | *moaC* | Molybdopterin cofactor synthesis protein C | -1.4 | 6.4E-04 | |  |
| cg0282 |  | CsbD family protein probably involved in stress response | -1.9 | 5.7E-06 | |  |
| cg0337 | *whiB4* | Putative transcriptional regulator, WhiB-family | -1.1 | 2.3E-06 | |  |
| cg0562 | *nusG* | Transcription antitermination protein NusG | -1.1 | 1.3E-02 | |  |
| cg0564 | *rplA* | 50S ribosomal protein L1 | -1.1 | 2.2E-03 | |  |
| cg0612 | *dkg* | Putative aldo/keto reductase, related to diketogulonate reductase | -1.2 | 6.2E-04 | |  |
| cg0780 |  | Membrane protein, ribonuclease BN-like family | -1.5 | 8.2E-05 | |  |
| cg0797 | *prpB1* | Putative (methyl)isocitrate lyase | -1.5 | 1.9E-04 | |  |
| cg0798 | *prpC1* | Putative (methyl)citrate synthase | -1.5 | 2.7E-05 | |  |
| cg1106 |  | conserved hypothetical protein | -2.0 | 2.8E-03 | |  |
| cg1236 | *tpx* | Thiol peroxidase | -1.9 | 1.9E-03 | |  |
| cg1409 | *pfkA* | 6-Phosphofructokinase | -1.6 | 9.3E-04 | |  |
| cg1512 |  | Hypothetical protein | -2.6 | 3.2E-03 | |  |
| cg1642 |  | Siderophore-interacting protein | -2.4 | 2.4E-03 | |  |
| cg2191 |  | Conserved hypothetical protein | -1.3 | 3.3E-03 | |  |
| cg2250 |  | Putative secreted protein | -1.4 | 1.4E-03 | |  |
| cg2320 |  | Putative transcriptional regulator, ArsR-family | -1.6 | 4.7E-03 | |  |
| cg2411 |  | Conserved hypothetical protein, HesB/YadR/YfhF family | -1.1 | 2.3E-02 | |  |
| cg2451 |  | Conserved hypothetical protein | -2.2 | 2.7E-05 | |  |
| cg2510 | *bex* | Putative Bex protein, GTP-binding protein ERA-like | -1.3 | 1.0E-03 | |  |
| cg2554 | *rbsK2* | Ribokinase | -1.0 | 6.9E-05 | |  |
| cg2591 | *dkgA* | Putative 2,5-diketo-D-gluconic acid reductase | -2.1 | 4.8E-04 | |  |
| cg2782 | *ftn* | Ferritin-like protein | -2.4 | 1.9E-03 | |  |
| cg2833 | *cysK* | Cysteine synthase | -1.4 | 1.2E-05 | |  |
| cg2891 | *pqo* | Pyruvate:quinone oxidoreductase | -1.4 | 3.8E-06 | |  |
| cg2957 |  | Conserved hypothetical protein | -1.1 | 5.7E-03 | |  |
| cg3264 |  | Conserved hypothetical protein | -1.8 | 9.9E-05 | |  |
| cg3299 | *trxB1* | Thioredoxin (TRX) | -1.8 | | 2.2E-05 | |

^a^ Gene ID, gene name and function of proteins are given according to CoryneRegNet (http://coryneregnet.compbio.sdu.dk/v6/index.html) and from previous studies. ^b^ Relative RNA amount of *sigA* overexpressing strain against the control strain with the empty vector was shown as log 2 values (M-values). ^c^ FDR represents false discovery rate. 50 µM IPTG were added from the beginning of the cultivation.
